# Drivers' decision-making when attempting to cross an intersection results from choice between affordances

**DOI:** 10.3389/fnhum.2014.01026

**Published:** 2015-01-09

**Authors:** Geoffrey Marti, Antoine H. P. Morice, Gilles Montagne

**Affiliations:** Aix-Marseille Université, CNRS, ISM UMR 7287, 13288Marseille, France

**Keywords:** affordances, intersection crossing, crossing possibilities, stopping possibilities, virtual reality

## Abstract

In theory, a safe approach to an intersection implies that drivers can simultaneously manage two scenarios: they either choose to cross or to give way to an oncoming vehicle. In this article we formalize the critical time for safe crossing (*CT*_*cross*_) and the critical time for safe stopping (*CT*_*stop*_) to represent crossing and stopping possibilities, respectively. We describe these critical times in terms of affordances and empirically test their respective contribution to the driver's decision-making process. Using a driving simulator, three groups of participants drove cars with identical acceleration capabilities and different braking capabilities. They were asked to try to cross an intersection where there was an oncoming vehicle, if they deemed the maneuver to be safe. If not, they could decide to stop or, as a last resort, make an emergency exit. The intersections were identical among groups. Results showed that although the crossing possibilities (*CT*_*cross*_) were the same for all groups, there were between-group differences in crossing frequency. This suggests that stopping possibilities (*CT*_*stop*_) play a role in the driver's decision-making process, in addition to the crossing possibilities. These results can be accounted for by a behavioral model of decision making, and provide support for the hypothesis of choice between affordances.

## Introduction

Driving a car is a typical situation in which an agent has to choose the most suitable maneuver to perform from multiple alternatives. For instance, when approaching an intersection, a driver has to decide whether to cross (before the road is blocked by oncoming traffic) or to stop. In this article, we argue that this real-life driving situation can be used as a paradigm to investigate a specific facet of decision making assuming that choice has to be performed between co-existing affordances. As an example of the hypothesis, imagine yourself driving a car that is not the one you normally use. If the acceleration of both cars is identical, then there is no apparent reason to change the way you approach the intersection, as the opportunity to successfully cross the intersection depends on (among other things) the car's acceleration. However, would you cross the intersection in the same way if the two cars had different braking capabilities? Probably not. It can therefore be argued that drivers have to choose between possibilities for crossing and stopping. We assume that the decision-making process at the approach to an intersection would initially consists in perceiving which maneuvers are possible and which are not, and secondly in selecting the most suitable maneuver among available possibilities. Is this decision a function of the relative chance of success of each maneuver, the fit between maneuver and the agent's aims and/or the risk they pose to the driver's life?

On the one hand, the perception of whether a behavior is possible or not is central to the driver's safety. For instance, an attempt to cross the intersection could prove fatal if the driver misjudges the acceleration of their car and the opportunity to cross before oncoming traffic arrives. The ability of an agent to perceive “action-scaled” affordances (the potential for action defined by the environment with respect to the agent's ability to take action) has received increasing attention since Gibson's formulation of the theory of affordances (Gibson, 1977). Previous work has investigated perception of the “pass-ability” of a shrinking gap between converging obstacles (Fajen and Matthis, 2011) and the “avoid-ability” of a collision by braking (Fajen, 2005a,b,c; Fajen and Devaney, 2006). These studies have shown that relevant properties of the environment are perceived in relation to the kinematic characteristics of the person's body or vehicle. From this perspective, the selection of the appropriate action entails scaling environmental properties (e.g., the deceleration required to stop before hitting an obstacle) to the action capabilities of the driver-car dyad (e.g., their maximum deceleration).

On the other hand, the selection of one solution at the expense of other alternatives is central to goal satisfaction. For instance, if the driver is unable to cross an intersection, he/she must select a second possible maneuver. The mechanisms that underlie the selection of the final decision have not been elucidated (see Michaels, 2003 concerning debates in ecological psychology). They may relate to their respective “valence” (Lewin et al., 1936), or whether possible solutions are attractive or repulsive in terms of behavioral dynamics (Warren, 1998, 2006). Both of these perspectives imply that potential solutions have a positive or negative meaning for the agent depending on their goal.

Unfortunately, few experimental studies have investigated action selection and affordance perception when different opportunities for action are available at the same time. Mark and colleagues (Mark et al., 1997; Gardner et al., 2001; see also Mantel et al., 2012 for a sketch of behavioral organization in presence of coexisting action modes) explored how behavior is constrained in presence of multiple affordances. They showed that when the goal achievement (i.e., reaching an object) is not constrained by a particular action mode (e.g., extending only the arm or combining the use of arm plus torso and/or shoulder), the critical boundary at which participants naturally switch from an action mode to another may change depending on postural constraints, accuracy requirements and comfort. However, this study not only focuses on body-scaled affordances but is also restricted to the choice among several affordances for the achievement of a unique goal. Ye et al. (2009) went further in the multiple affordances issue by investigating choice among several objects affordances that satisfy various goals. They evidenced that the perception of an object's affordance affects the detection of the other affordances offered by that object. Therefore, this study completes the need of empirical investigation of how choices are made among available affordances (see Stoffregen, 2003, 2004 for a definition of the affordance concept entailing a choice between a large amount of affordances in the world). However, the aforementioned studies do not consider some features of the intersection crossing situation. Indeed, such a paradigm implies a choice between action-scaled affordances (e.g., crossing vs. stopping opportunities scaled in reference with action capabilities) that concern antagonist goals (e.g., time saving vs. safe keeping).

Driver behavior at intersections has received a lot of attention in the literature. Research has been carried out to identify the ability of a stationary driver to detect whether there is a safe gap to allow crossing between oncoming vehicles (See Caird and Hancock, 2002 for a review). Lee et al. (1984) were the first to our knowledge to take advantage of the affordance framework in order to investigate the decision to cross or not in situations where perception and action are coupled. They showed in a task consisting in crossing or not by walking on a road through gaps in traffic that adults accepted smaller gaps than children. Since perceiving the affordance of gap in traffic entails perceiving the gap with reference to the crossing time capabilities, the children's cautious behavior was attributed to their inconsistency in walking at a constant speed through gaps. Plumert et al. (2011) moreover evidenced in an intersection crossing task with bicycle that the size of accepted gap tended to decrease with exposure. However, these tasks do not entail managing simultaneously the crossing and the stopping possibilities since walking and cycling allowed instantaneous stopping in front of the traffic and also waiting for an acceptable gap. As far as we know, requiring an agent to choose between crossing, stopping or even turning around to avoid a collision at an intersection does not seem to have been investigated experimentally. Nevertheless, it is a highly relevant paradigmatic task for understanding the decision-making process.

Gibson and Crooks (1938) proposed a theoretical framework capable of explaining driver's behavior in such a situation. In this seminal paper, they defined two major concepts: the Field of Safe Travel (FST) defined as the “*field of possible paths which the car may take unimpeded*” (p. 454), and the Minimum Stopping Zone (MSZ) defined as the “*zone within which our driver could stop if he had to”* (p. 457). Safe driving behavior was hypothesized to be the driver's response to the simultaneous perception of the boundaries of these two spatiotemporal regions, which would specify the driver's current crossing and stopping possibilities. Gibson and Crooks proposed that stopping-ability depends on the relation between FST and MSZ. They suggested that such an affordance can be formalized as the ratio between the FST and the MSZ. This attention prefigures a great tradition in affordance research about how to formalize an affordance (e.g., see Warren, 1984; Shaw et al., 1995 pioneering work on affordance modeling). According to this “field-zone ratio”, drivers take into account all safe paths and behave in such a way that the set of positions predicted by emergency braking is located in this field. However, Gibson and Crooks did not explicitly consider crossing-ability affordance and thus did not address the issue of what could be the driver's behavior when both crossing and stopping actions are afforded.

In the present study, we argue that it may be possible to provide a complete account of drivers' decision-making process in terms of choice between two or more co-existing possibilities. First, we formalized critical times at which safe crossing and safe stopping are no longer possible, based on Gibson and Crooks' theoretical description of the FST and the MSZ. Hereafter, we denote these critical times *CT*_*cross*_ and *CT*_*stop*_, respectively. Then, we investigated empirically whether and how these times influence driving behavior at an intersection. We designed a task where the driver's main goal is to safely cross an intersection. This task required managing simultaneously the crossing and the stopping possibilities unlike previously mentioned studies which only entailed managing crossing possibilities (Lee et al., 1984; Plumert et al., 2011). It is consequently assumed that the drivers will either try to cross or stop depending on their capabilities. We predict that if different possibilities for actions are simultaneously available, the chance of success of each action will influence the driver's final decision. Specifically, drivers approaching an intersection were asked to decide whether to cross or stop in situations where crossing conditions were the same, but stopping conditions were different. We predicted that in this scenario, the driver's decision about whether to cross would be different.

## Methods

### Participants

Thirty experienced drivers (12 female, 18 male, aged 25.8 ± 2.8 years), who held a valid driver's license gave their informed written consent to participate to this experiment. They all had normal or corrected-to-normal vision and were not informed about the purpose of the study. A local ethics committee approved the experiment.

### Apparatus

**Figure 1:**
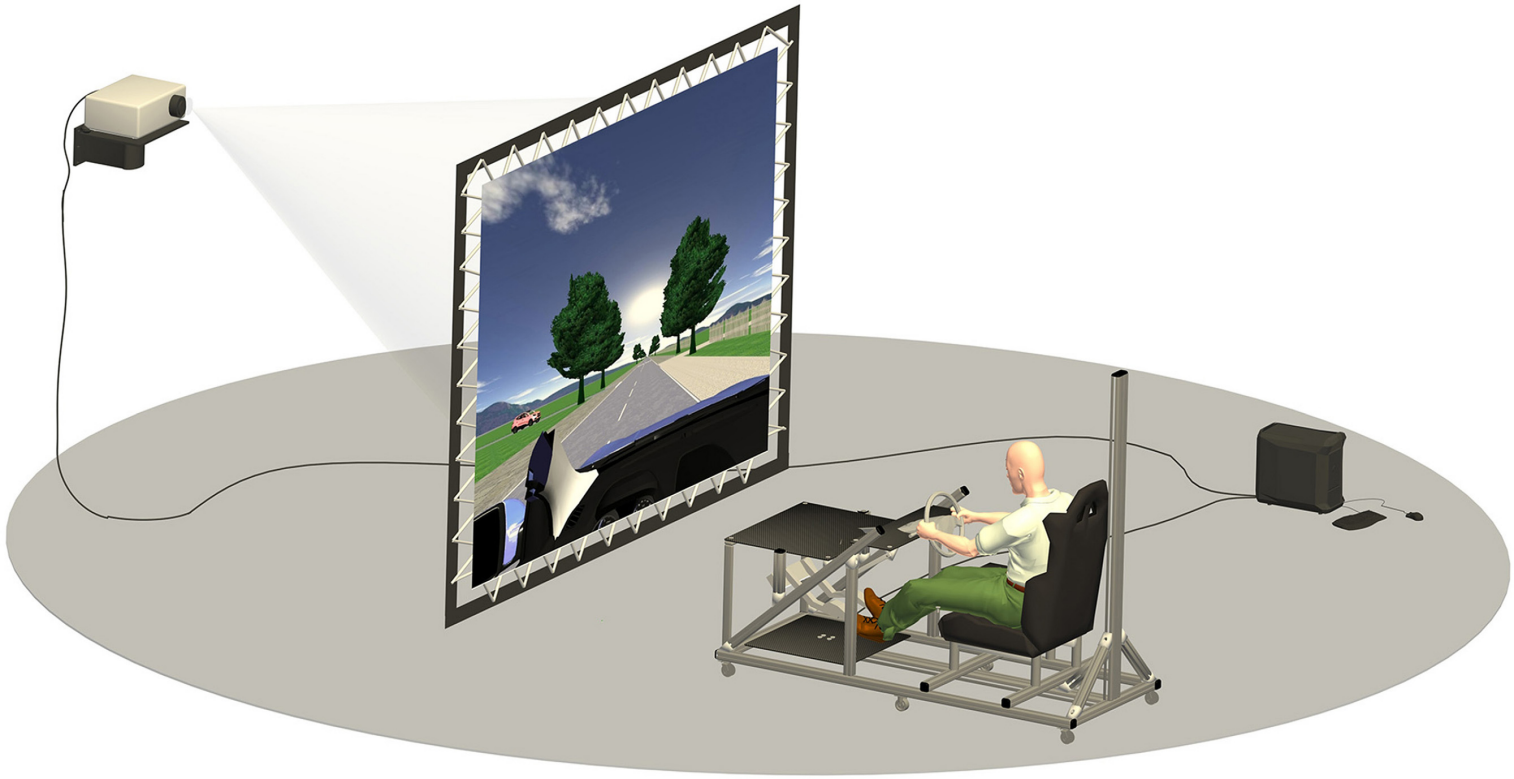
**Overview of the driving simulator and the virtual scene**. Participants are seated in a driving simulator in front of a large screen (2.3 × 3 m) and interact with intersection crossing situations in a virtual reality environment. They were instructed to try to cross the intersection before the car approaching from the left by accelerating. Alternatively, they could stop before the intersection by braking, or as a last resort they could exit into an emergency lane.

Figure 1 is an overview of the experimental setup. Participants sat in a fixed-base driving simulator (Mobsim, France). Their eye level was 1.1 m above the floor and 1.5 m in front of a large screen (2.5 × 3.2 m), which encompassed 94° of their horizontal field of view. The action of their right foot on the brake and accelerator pedals and their manipulation of the steering wheel (ECCI Trackstar 6000 GTS) were monitored via a USB signal sent to a host computer (Intel® Core™ i7-950 Processor @3.07 GHz; NVIDIA® GeForce® GTX 580 graphics card). Customized OpenGL virtual reality software updated the virtual scene. The virtual scene was rear-projected onto the screen by a video projector (Barco iQ R500) at a frame rate of 60 Hz with a resolution of 1152 × 864 pixels. No speedometer or audio feedback was provided to participants.

A 20% deviation of the accelerator or brake pedal from the neutral position constituted a single constant maximum acceleration or deceleration of the virtual car, respectively until the end of the trial. A 45° clockwise rotation of the steering wheel initiated a single lateral exit of the participant's car onto the roadside, followed by an immediate stop. Participants were only able to select one, unique action (i.e., maximum acceleration, maximum braking or a roadside exit) during the course of one trial. Although it was impossible for participants to control the maneuver once it has been initiated, the experimental setup reproduced real-life constraints in emergency situations where the action initiation time is crucial.

### Virtual world

From the participant's viewpoint, the visual scene was composed of an intersection formed by two straight, orthogonal roads, the cockpit of the virtual car, an oncoming car traveling orthogonally to participant's displacement and a blue sky (Figure 1). The driving environment consisted of a conventional two-lane, 7-m wide cement road in a flat rural environment. The right-hand side of lanes was delimited by a continuous white line and the left by a discontinuous white line. A 3.5-m wide gravel bank and a 3.5-m wide pavement bordered by trees were displayed on the right- and left-hand sides of the road, respectively. The participant's lane was crossed orthogonally by a 3.5-m wide single lane road bordered by trees. An oncoming vehicle approached the intersection on this road. A white horizontal line and two checkerboard panels attached to poles marked the beginning of the experimental space. The participant's car and the oncoming car were displayed as 3D blue and red models of a 4 × 4 Hyundai ix35, respectively. Both cars were 4.41 m long, 2.13 m wide, and 1.68 m high.

### Procedure

Participants were divided into three groups according to the maximum deceleration of the virtual car: 5 m/s^2^ D_max_ (the low group), 10 m/s^2^ D_max_ (the high group) and ∞ m/s^2^ D_max_ (the inf group, able to stop instantaneously). The experiment was divided into four phases: deceleration calibration, acceleration calibration, familiarization and the experimental phase. At the beginning of each phase, participants automatically moved through an empty rural environment at a constant velocity along the longitudinal road axis for 5 s before the trial actually started. During this period, any pedal action by participants was ignored by the simulator. A digital 3 s countdown preceded the actual trial. At this point, the oncoming vehicle (or an immobile barrier, see the deceleration calibration in the next section) appeared and the velocity of the participant's car did not change unless they manipulated the pedals or the steering wheel. It is important to note that participants were not allowed to continuously regulate their displacement; they could only initiate either a maximum acceleration or deceleration. The trial ended when the participant's car was 10 m beyond the intersection or had stopped.

The deceleration calibration phase was designed to enable participants to calibrate the maximum deceleration (D_max_) of the virtual car. Participants were instructed to stop in front of a red and white barrier placed just before the intersection. Specifically, they were asked to initiate the driving maneuver with the maximum braking capabilities of their car at the last possible moment. The initial distance between the participant's car and the barrier, and the participant's initial velocity were manipulated to create six *CT*_*stop*_ values (depending on the group). *CT*_*stop*_ is defined as the last time to stop safely by braking at D_max_ (see the Supplementary Material for a mathematical definition, Independent Variables subsection and Table 1 for initial values). In sum, the participant's task was to brake at a time as close as possible to the *CT*_*stop*_. Conditions were allocated randomly and repeated twice. A trial was recorded as successful when the participant braked at least 0.55 s before the *CT*_*stop*_ or at most 0.20 s after it. A braking time of *t* = *CT*_*stop*_ – 0.55 s meant that they had stopped 14 m before the barrier (on average). Conversely, a maneuver initiated at *t* = *CT*_*stop*_ + 0.20 s meant that they had stopped 5 m after it (on average). The superior tolerance was weaker than the inferior tolerance in order to discourage participants from braking after the *CT*_*stop*_ as this decision led to a collision.

**Table 1:** **Initial kinematics of intersection-crossing situations during calibration and experimental phases**.

**Phase**	**Stopping possibilities**			**Intersection-crossing situation**						**Crossing possibilities**
	***CT_*stop*_* (s)**			**Driver's car initial kinematics**			**Oncoming car initial kinematics**			***CT_*cross*_* (s)**
	**Low**	**High**	**Inf**	**TTC_s_ (s)**	**Distance (m)**	**Velocity (m/s)**	**Distance (m)**	**Velocity (m/s)**	**TTC_o_ (s)**	**All groups**
Calibration	1.00	2.00	3.00	3.00	63.40	20.00	42.61	12.88	3.19	1.50
	1.45	2.25	3.05	3.05	52.10	16.00	34.70	10.03	3.31	1.75
	1.40	2.50	3.60	3.60	82.50	22.00	55.97	14.49	3.76	2.00
	1.36	2.75	4.16	4.16	119.70	28.00	82.00	18.94	4.25	2.25
	1.25	3.00	4.75	4.75	169.50	35.00	116.85	24.09	4.79	2.50
	1.65	3.25	4.85	4.85	158.50	32.00	109.15	21.92	4.91	2.75
Experiment	-1.50	0.50	2.50	2.50	103.30	40.00	70.53	27.52	2.51	0.00
	-1.50	0.50	2.50	2.50	103.30	40.00	70.53	26.22	2.63	1.50
	-1.50	0.50	2.50	2.50	103.30	40.00	70.53	25.91	2.66	2.50
	-0.50	1.00	2.51	2.51	78.30	30.00	53.04	20.53	2.51	0.00
	-0.50	1.00	2.51	2.51	78.30	30.00	53.04	19.27	2.67	1.50
	-0.50	1.00	2.51	2.51	78.30	30.00	53.04	18.96	2.72	2.50
	0.45	1.50	2.55	2.55	56.80	21.00	38.00	14.30	2.55	0.00
	0.45	1.50	2.55	2.55	56.80	21.00	38.00	13.11	2.78	1.50
	0.45	1.50	2.55	2.55	56.80	21.00	38.00	12.78	2.85	2.50
	0.25	2.00	3.75	3.75	134.60	35.00	92.43	25.43	3.57	0.00
	0.25	2.00	3.75	3.75	134.60	35.00	92.43	23.99	3.79	1.50
	0.25	2.00	3.75	3.75	134.60	35.00	92.43	23.41	3.88	2.50
	1.55	2.50	3.46	3.46	69.00	19.00	46.53	13.85	3.25	0.00
	1.55	2.50	3.46	3.46	69.00	19.00	46.53	12.58	3.58	1.50
	1.55	2.50	3.46	3.46	69.00	19.00	46.53	12.09	3.72	2.50
	1.75	3.00	4.25	4.25	109.60	25.00	74.94	18.80	3.90	0.00
	1.75	3.00	4.25	4.25	109.60	25.00	74.94	17.40	4.22	1.50
	1.75	3.00	4.25	4.25	109.60	25.00	74.94	16.79	4.37	2.50

*Intersection distances, velocities and time to contact referred to the intersection (TTC*_s_ *and TTC_o_) are reported for both participant and oncoming cars. CT_*cross*_ and CT_*stop*_ values are computed from the maximum acceleration and braking capabilities of the driven car and from the initial kinematics of the oncoming and participant cars*.

The acceleration calibration phase was designed to enable participants to calibrate the maximum acceleration (A_max_) of their virtual car. Participants were instructed to cross the intersection before an oncoming car moving at a constant velocity reached the intersection and blocked the crossing. Thus, drivers had to initiate the driving maneuver with the maximum acceleration capabilities of their car at the last possible moment. Their distance to the intersection and their initial velocity, and the distance between the oncoming car and the intersection and its velocity were manipulated to create six *CT*_*cross*_ values (the same for each group). *CT*_*cross*_ is defined as the last time to cross safely the intersection by accelerating at A_max_ (see the Appendix for a mathematical definition, Independent Variables subsection and Table 1 for initial values). Here, the participant's task was to accelerate as close as possible to *CT*_*cross*_. These conditions were randomly allocated and repeated twice. A trial was recorded as successful when the participant decided to accelerate at a time at least 0.55 s before the *CT*_*cross*_ or a maximum of 0.20 s after it. If participants decided to accelerate at *t* = *CT*_*cross*_ – 0.55 s, they crossed the intersection before the oncoming obstacle with a safety margin of 8 m (on average). Conversely, if they initiated the maneuver at a time later than *t* = *CT*_*cross*_ + 0.20 s, they collided with the oncoming car as the average safety margin was –0.5 m.

In each calibration trial, success was indicated to the participant by a green symbol and failure by a red symbol. Participants performed 12 trials in each calibration sub-phase. The 12-trial sequence ended when the participant had successfully completed at least eight trials. Otherwise, the 12-trial sequence was repeated until they met this criterion.

During the experimental phase, an oncoming car moved orthogonally at a constant velocity (see Table 1 for velocity values) toward the intersection and stopped in the participant's lane, blocking the road. In each trial, participants had to decide whether to cross, stop or exit. They could either try to cross the intersection before the oncoming car arrived by initiating a maximum acceleration before it was too late to cross safely (i.e., before the *CT*_*cross*_). Otherwise, they could decide to stop before they reached the intersection by initiating a braking maneuver before it was no longer possible to stop safely (i.e., before the *CT*_*stop*_). As a last resort, they could decide to bail out by steering into the emergency lane to ensure their safety. In any given trial, they selected one of these three actions, which were indicated in real time by “GO,” “STOP,” and “OUT” messages displayed on the screen to provide an augmented feedback about the maneuver selected. At the end of each trial a green symbol informed the participants that they had not collided with the oncoming car, while a red symbol indicated a collision. No feedback was provided when participants bailed out; this was to discourage them from abusing this emergency alternative. The entire experiment lasted approximately one and a half hours. A short familiarization phase preceded the experimental phase. In this phase, the participant was exposed once to the 18 experimental conditions.

### Independent variables

**Figure 2:**
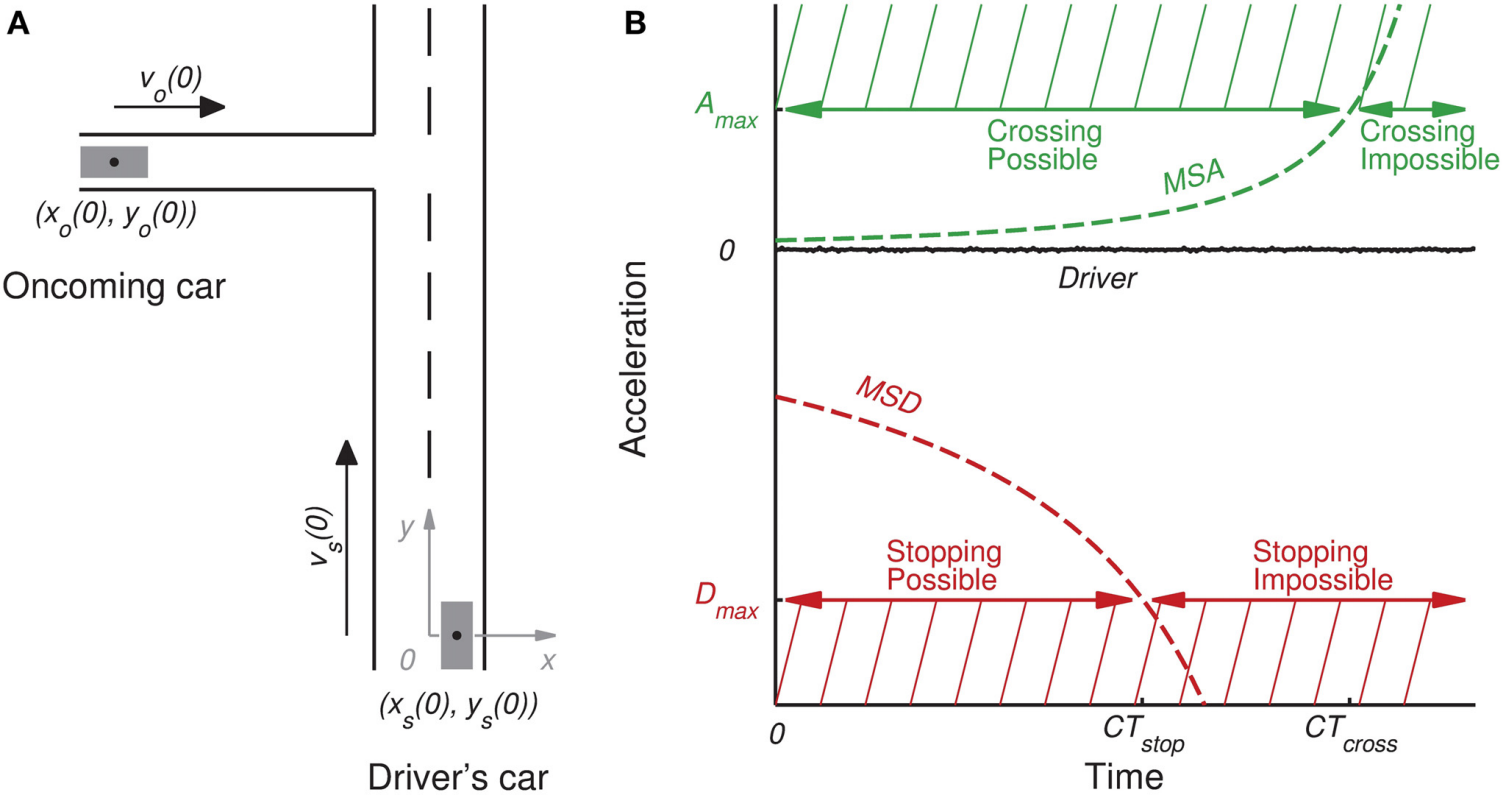
**A geometric illustration of the intersection at the beginning of the trial (A), and relevant variables plotted over time (B)**. **(A)** The left-hand side illustrates a typical intersection encountered by the driver at the beginning of the trial (*t* = 0). The 2D coordinates of the participant (*x*_*s*_(0), *y*_*s*_(0)) and the oncoming car (*x*_*o*_(0), *y*_*o*_(0)) refer to the center of the vehicles (black dot). The arrows indicate the direction of movement and its sizes represent the relative velocities of the participant and the oncoming cars (denoted *v*_*s*_ (0) and *v*_0_ (0), respectively). **(B)** The right-hand side illustrates the evolution of the minimum satisfying acceleration to safely cross the intersection (MSA, green line) and the minimum satisfying deceleration to stop just before it (MSD, red line) if the driver does not initiate any action. The participant's car has a maximum acceleration capability A_max_ (green hatched area) and a maximum deceleration capability D_max_ (red hatched area). These capabilities define the limits of the driver's possibilities. Crossing possibilities are specified by *CT*_*cross*_, the critical time at which the MSA exceeds the participant's A_max_. Stopping possibilities are specified by *CT*_*stop*_, the critical time at which the MSD exceeds the participant's D_max_.

The independent variables and their respective contribution to the success of participant's maneuver are illustrated in Figure 2. The maximum acceleration of the virtual car (A_max_) was set to 2 m/s^2^ for all groups. The maximum deceleration (D_max_) was a between-group variable. As mentioned above, participants were divided into three groups according to the maximum deceleration of the virtual car: 5 m/s^2^ D_max_ (the low group), 10 m/s^2^ D_max_ (the high group) and ∞ m/s^2^ D_max_ (the inf group). As the inf group was able to stop instantaneously at any given moment, they only had to decide whether to cross. Consequently, differences in the behavior of the inf group and the other groups made it possible to compare the influence of a single cross-ability affordance and choice between cross-ability and stop-ability affordances.

The *CT*_*cross*_ (i.e., the critical time at which the minimum satisfying acceleration to safely cross the intersection, denoted MSA, exceeds the driver's maximum acceleration capabilities A_max_) and the *CT*_*stop*_ (i.e., the critical time at which the minimum satisfying deceleration to safely stop, denoted MSD, exceeds the driver's maximum braking capabilities D_max_) were between-trials variables. The computations of these variables are defined in the Supplementary Material.

The experimental conditions were created by adjusting the extrinsic features of the intersection between trials. Hence, we manipulated the distance from the drivers to the intersection and their initial velocity, and the distance between the oncoming car and the intersection and its velocity for each of the three groups (Table 1). This created 18 intersection-crossing combinations, which were randomly sorted and repeated five times. These combinations resulted in three *CT*_*cross*_ values (the latest time it was possible to cross safely by accelerating at A_max_) that were applied to each group (0.00, 1.50, and 2.50 s), and six *CT*_*stop*_ values (the latest time it was possible to stop safely by braking at D_max_) that ranged from −1.50 to 4.25 s depending on the braking capability of the virtual car (the ranges were different for each group). The ratio between the initial distance of the oncoming car and the initial distance of the participant's car was adjusted so that the oncoming car was always initially located 35° to the left of the participant's field of view.

*CT*_*cross*_ and *CT*_*stop*_ values specified the last moment (relative to the beginning of the trial) at which the maximum acceleration and deceleration had to be initiated in order to cross or stop safely, respectively. Therefore, from a functional point of view, positive, negative and null values of *CT*_*cross*_ and *CT*_*stop*_ indicated safe crossing and stopping after, before and at the beginning of the trial, respectively. For example, a *CT*_*stop*_ equal to 2.50 s meant that the driver could initiate a deceleration at a maximum of 2.50 s after the beginning of the trial (i.e., later than in a trial with a *CT*_*stop*_ value equal to 1.50 s). Conversely, a *CT*_*stop*_ value equal to −1.50 s meant that at the beginning of the trial, it was already impossible to stop safely (by 1.50 s).

### Dependent variable

The dependent variable was the decision selected by each participant (i.e., to cross, stop, or to exit), the time at which the pedal corresponding to the selected decision was pushed and the number of collisions with the oncoming car. We computed the individual frequency of each decision as the ratio of the number of decision types over the number of trials and focused on crossing frequency as a function of *CT*_*cross*_ and *CT*_*stop*_ conditions. We computed the individual average decision time for the overall trials (independently of the occurrence of collisions).

### Predictions

Our initial hypothesis was that the selection of driving maneuvers was driven by the perception of the corresponding affordance. In other words, crossing frequency would increase with the viability of crossing possibilities and stopping frequency would increase with the viability of stopping possibilities. Finally, drivers were expected to exit the road when it was not possible to either cross or stop.

Based on the frequency of crossing maneuvers, we tested the hypothesis of the choice between cross-ability and stop-ability affordances when drivers have to select the best maneuver at the approach to an intersection. From an extrinsic viewpoint, all groups of drivers experienced identical intersection-crossing situations. However, from an intrinsic viewpoint, stopping possibilities (*CT*_*stop*_) were manipulated between groups by varying maximum braking capabilities (D_max_). Crossing possibilities (*CT*_*cross*_) were not manipulated between groups as maximum acceleration (A_max_) was kept constant. Therefore, if the stop-ability affordance played a role in decision-making, the manipulation of D_max_was expected to affect the average crossing frequency. More specifically, the low D_max_ group was expected to cross the intersection at a higher frequency on average than the high D_max_ group because the former had lower braking capabilities.

We investigated the choice between affordances hypothesis in more detail by examining, for each group, the effect of *CT*_*cross*_ and *CT*_*stop*_. As described earlier, the three *CT*_*cross*_ values (0.00, 1.50, and 2.50 s) were combined with six different *CT*_*stop*_ values, yielding a total of 18 intersection-crossing situations. Depending on the intersection-crossing situation and D_max_, drivers encountered *CT*_*stop*_ values ranging from −1.50 to 1.75, 0.50 to 3.00, and 2.50 to 4.25 s for the low, high and inf groups, respectively. We predicted that in the inf group (that only had to handle the cross-ability affordance, given that drivers could stop instantly) crossing frequency would increase with *CT*_*cross*_, while *CT*_*stop*_ would have no effect. For the low and high groups, we expected to see a combined effect of *CT*_*cross*_ and *CT*_*stop*_ on the average crossing frequency. This would demonstrate that *CT*_*cross*_ and *CT*_*stop*_, as formalized in this experiment, contribute to choice between affordances. We expected that the crossing frequency would increase with increasing *CT*_*cross*_ in the same way for each group, and would increase with decreasing *CT*_*stop*_, more so for the low group exposed to low *CT*_*stop*_ values (*CT*_*stop*_ = 0.33 s on average) than for the high group exposed to higher *CT*_*stop*_ values (*CT*_*stop*_ = 1.75 s on average).

## Results

### Calibration

Our first analysis checked that all groups had successfully calibrated their virtual maximum deceleration (D_max_) and acceleration (A_max_) during the calibration phase. Drivers were assumed to have calibrated their A_max_ and D_max_ when they succeeded in 8 out of 12 trials in acceleration and braking calibration tasks. We analyzed the number of 12-trial sequences required to achieve this criterion and the overall success rate for all groups. Participants in the three groups calibrated D_max_ in 2.30 ± 0.95, 1.90 ± 0.88, and 1.10 ± 0.32 sequences of 12 trials with a final success rate equal to 9.40 ± 1.17, 9.00 ± 0.94, and 10.50 ± 1.08 trials, for the low, high and inf groups, respectively. Participants calibrated A_max_ in 3.00 ± 1.05, 3.00 ± 1.70, and 3.50 ± 1.08 sequences of 12 trials with a success rate in the last 12-trial sequence equal to 9.10 ± 1.79, 8.80 ± 0.92, and 9.10 ± 1.37 trials, for the low, high and inf groups, respectively. In sum, participants quickly calibrated their maximum action capabilities. The calibration time for D_max_ increased as D_max_ decreased, while the calibration time for A_max_ was constant across groups. Finally, the similarity in the final success rates achieved by all groups suggests that all participants had a similar level of competence before the experimental phase.

### Collisions

Our analyses then investigated differences in collision frequency according to group and decision type. Despite the similar success rates at the end of the crossing and stopping calibration phases, the three groups of participants collided with the oncoming car at different frequencies (24.56 ± 5.25, 14.00 ± 3.40, and 8.22 ± 4.92% for the low, high and inf groups, respectively). Moreover, the decision to cross led to more collisions (17.00 ± 4.69, 9.22 ± 3.78, and 8.22 ± 4.92%) than the decision to stop (7.56 ± 3.66, 4.78 ± 2.35, and 0% for the low, high and inf groups, respectively). Overall, these results show that the risk of collision due to the decision to cross increased with a decrease in maximum braking capabilities.

### Decision time

Thirdly, analyses aimed at investigating the changes in driver's decision time depending on groups, decision type and intersection-crossing situations. Decision time did not appear to differ between the low and high groups (0.81 ± 0.20 and 0.91 ± 0.21 s) but increased for the inf group (1.53 ± 0.42 s). Moreover, decision time for crossing, stopping and exiting (0.78 ± 0.27, 1.29 ± 0.61 and 1.48 ± 0.47 s, respectively) was in most cases in an ascending order (consistent with instructions), with the inf group showing a large tendency to decide stopping later than crossing. This suggests that the participants of the inf group exploited their braking possibility as a last resort, in opposition to the other groups that had to choose between affordances.

We further focused on the influence of intersection-crossing situations on the average decision time. We noted that *CT*_*stop*_ and TTC_s_ (Time to Contact of the driver's car referred to the intersection) co-varied in our intersection-crossing situations (Table 1). Their respective contributions on decision time was thus assessed for the three groups with comparisons of percentage of variance of decision time provided by separate linear regressions between these two predictors and decision time, respectively. TTC_s_ was found to better account for the variations of decision time than *CT*_*stop*_ (85.54, 80.63, and 77.37% vs. 35.15, 65.59, and 77.37% the low, high and inf groups, respectively). The decision time increased with the increase of TTC_s_ (ranging from 0.66 to 1.05, 0.71 to 1.15, and 1.11 to 2.00 s for the low, high and inf groups, respectively). However, the influence of TTC_s_ was more important for the inf group than for the other groups. Finally, the decision time of the inf group decreased and was equal to 1.72, 1.49, and 1.37 s when *CT*_*cross*_ increased and were equal to 0.00, 1.50 and 2.50 s, respectively. On the contrary, the decision time remained quite constant for the low (0.87, 0.78, and 0.77 s) and high groups (0.99, 0.87, and 0.85 s) across manipulations of *CT*_*cross*_.

In sum, we reported that the decision time tends to decrease when *CT*_*cross*_ increased for the inf group. Moreover, decision time increased when TTC_s_ increased with a more important intensity for the inf group than for the other groups. The low and high group being limited in their maximum acceleration and deceleration capabilities took their behavioral decision earlier than the inf group which was only limited by a maximum acceleration.

### Decision frequency

**Figure 3:**
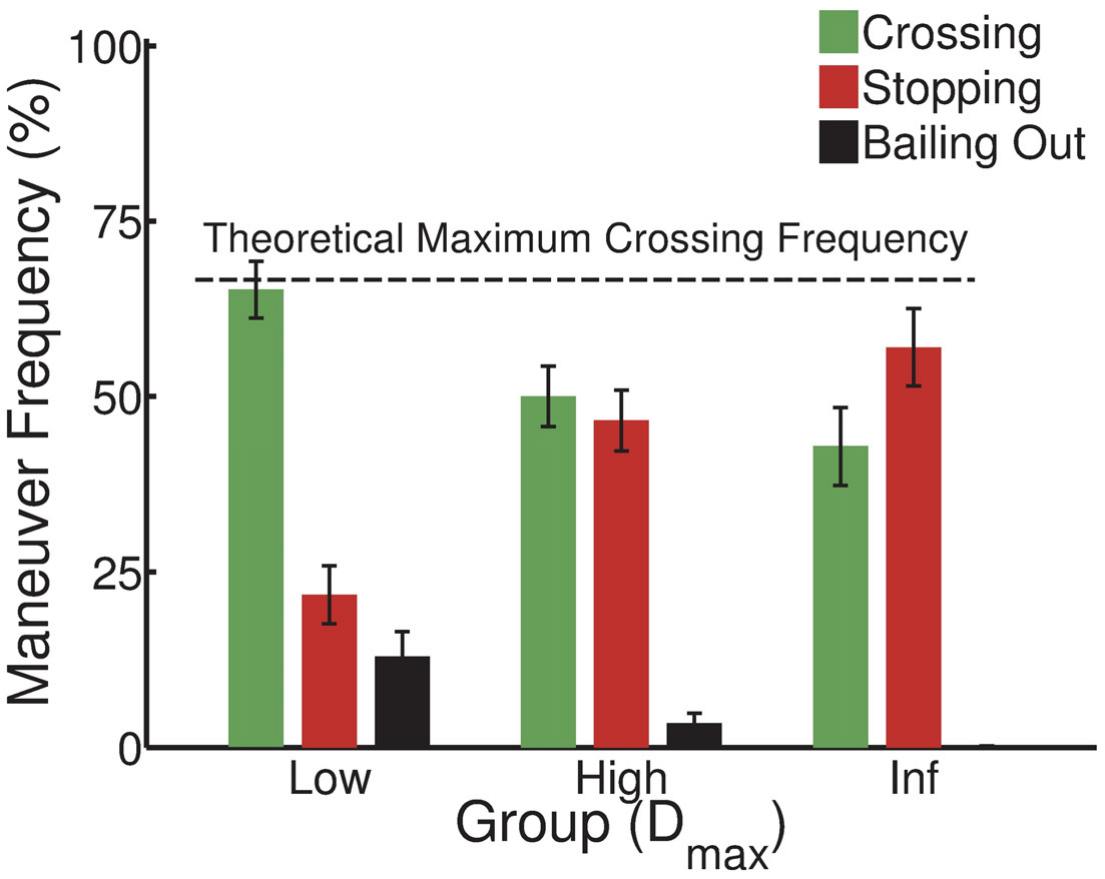
**Inter-individual average frequency of driving maneuvers (crossing, stopping and bailing out) for the low (D_max_ = 5 m/s^2^), high (D_max_ = 10 m/s^2^) and inf (D_max_ = ∞ m/s^2^) deceleration groups, respectively**. While the theoretical maximum crossing frequency corresponds to 12 out of the 18 trials (~66%) for which the initial *CT*_*cross*_ was positive for all groups, crossing frequencies decrease with the increase of stopping possibilities. Vertical bars indicate standard error of individual means.

The final and most important analysis investigated changes in the frequency of driving maneuvers across groups depending on their crossing and stopping possibilities during the experimental phase. Figure 3 shows that the frequency of all driving maneuvers (crossing, stopping and exiting) varied as a function of the between-group manipulation of D_max_. As deceleration capability (D_max_) increased, drivers attempted less often to cross the intersection (65.22 ± 12.78, 50.00 ± 13.66, and 42.89 ± 17.48% for the low, high and inf groups, respectively). In parallel, as D_max_ increased, drivers decided to stop more frequently (21.78 ± 13.12, 46.56 ± 13.75, and 57.00 ± 17.51% for the low, high and inf groups, respectively). Finally, as D_max_ increased, drivers exited less often (13.00 ± 11.10, 3.44 ± 4.64, and 0.11 ± 0.35% for the low, high and inf groups, respectively).

These differences in the frequency of stopping and exiting suggest a mixed perception of related affordances. The increase in the frequency of the decision to stop as D_max_ increased corresponded to an increase in stopping possibilities, as average *CT*_*stop*_ values increased with D_max_. Similarly, the decrease in the frequency of the decision to exit as D_max_ increased corresponded to a decrease in intersection-crossing situations where there were no crossing or stopping possibilities (i.e., *CT*_*cross*_ and *CT*_*stop*_ were less than or equal to zero at the start of the trial). Exiting maneuvers were observed in 13% of trials for the low group, while 11% of intersection-crossing situations prevented both safe crossing and stopping. On the other hand, the frequency of exiting in high and inf groups, which never encountered such situations, was close to zero.

However, the observed changes in crossing frequency with D_max_ suggested that there was a choice between a cross-ability affordance, directly related to the crossing action, and a stop-ability affordance that was *a priori* irrelevant to the decision to cross. As *CT*_*cross*_ conditions were identical for all groups, changes in crossing frequency as a function of *CT*_*cross*_ would tend to confirm that crossing possibilities influenced the decision to cross, independently of the group. In parallel, given that stopping possibilities were *a priori* only relevant to braking maneuvers and that they co-varied with D_max_, the D_max_ effect on crossing frequency would suggest that there is a choice between stopping and crossing possibilities when performing the crossing maneuver.

In order to investigate the choice between crossing and stopping affordances, a Two-Way ANOVA (*CT*_*cross*_ [0.00, 1.50, 2.50] × group [low, high, inf]) with *CT*_*cross*_ as a repeated measure was performed on crossing frequencies. *Post-hoc* comparisons were conducted using Newman–Keuls *a posteriori* tests. The ANOVA showed a significant main effect of *CT*_*cross*_ [*F*_(2, 54)_ = 67.16, *p* < 0.05]. *Post-hoc* analyses showed that crossing frequency significantly increased with each increase of *CT*_*cross*_ (*p* < 0.05). The ANOVA also showed a significant main effect of group [*F*_(2, 27)_ = 5.96, *p* < 0.05]. *Post-hoc* analyses showed that the crossing frequency was significantly higher for the low group than for the other groups. The group × *CT*_*cross*_ interaction was not significant [*F*_(4, 54)_ = 1.39, *p* > 0.05].

In brief, our results showed that crossing frequency increased with *CT*_*cross*_ for all groups. Between-group differences were observed in crossing frequency, although all groups experienced identical intersection-crossing situations and had identical maximum acceleration capabilities. Taken together, these results suggest that both crossing and stopping possibilities play a role in the decision to cross.

**Figure 4:**
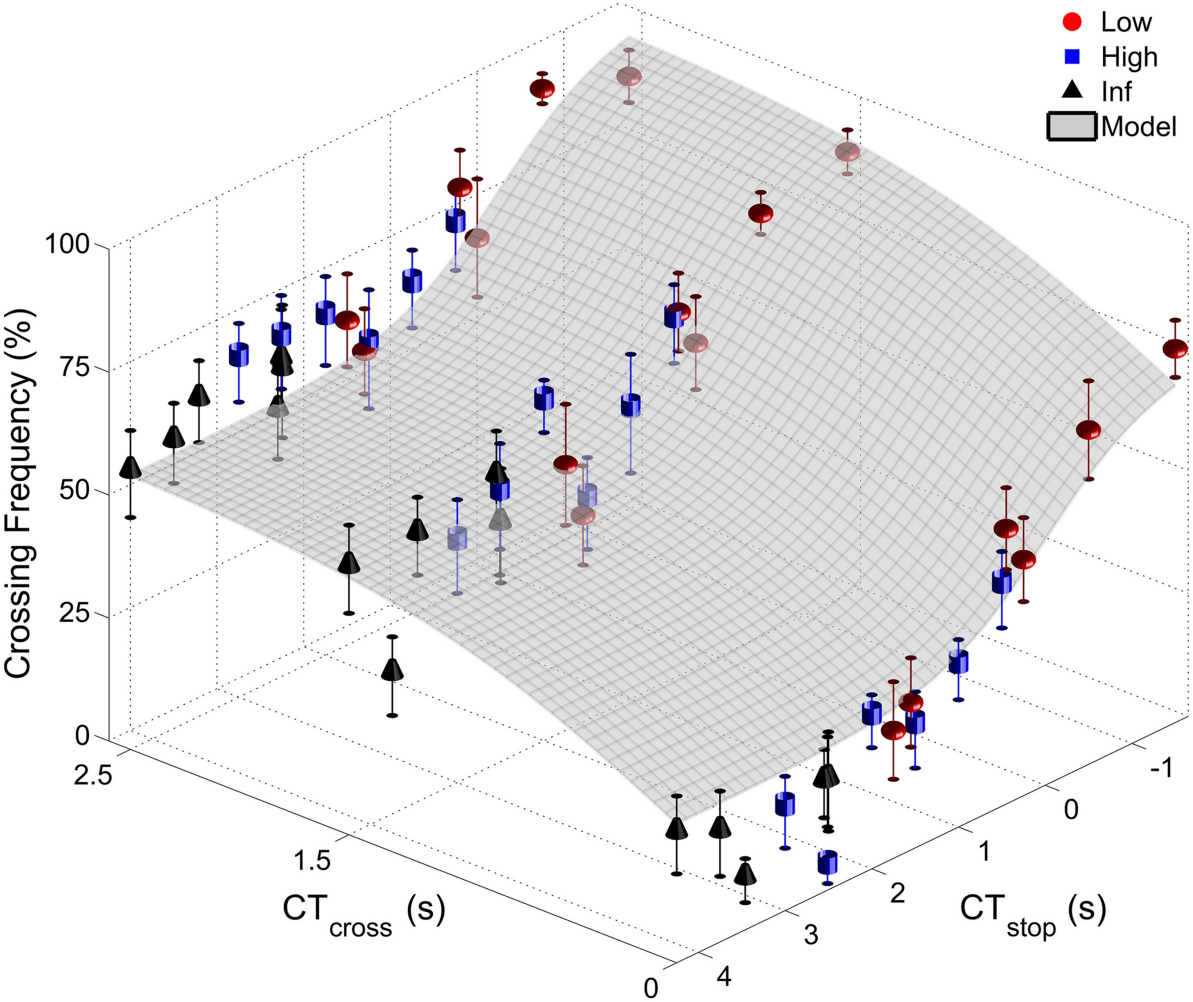
**Inter-individual average crossing frequency plotted as a function of *CT*_*cross*_ and *CT*_*stop*_ for the three groups (a circle, square, and a pyramid represent the low, high and inf groups, respectively)**. The exponential rise (gray curve) models the driver's tendency to cross the intersection as crossing possibilities (*CT*_*cross*_) increase and stopping possibilities (*CT*_*stop*_) decrease. Vertical bars show standard error of individual means.

As *CT*_*stop*_ co-varied with D_max_ and *CT*_*stop*_ conditions were different for each group (see Table 1), the influence of *CT*_*stop*_ can only be investigated by carrying out three separate Two-Way ANOVAs (*CT*_*cross*_ × *CT*_*stop*_) with repeated measures on *CT*_*cross*_ and *CT*_*stop*_ on crossing frequency. Figure 4 depicts the change in average crossing frequency as a function of *CT*_*cross*_ and *CT*_*stop*_. For each group, the ANOVAs again revealed a main effect of *CT*_*cross*_ [*F*_(2, 18)_ = 25.06; *F*_(2, 18)_ = 20.96 and *F*_(2, 18)_ = 25.85, *p* < 0.05 for the low, high and inf groups, respectively]. For all groups, *post-hoc* analyses revealed that the crossing frequency observed for *CT*_*cross*_ = 0 was significantly lower than that seen in the other *CT*_*cross*_ conditions (*p* < 0.05). The ANOVA also revealed a main effect of *CT*_*stop*_ for low and high groups [*F*_(5, 45)_ < 9.59; *F*_(5, 45)_ = 3.59, *p* < 0.05 for the low and high groups, respectively]. *Post-hoc* analyses showed that the crossing frequency displayed by the low group during the negative *CT*_*stop*_ conditions (*CT*_*stop*_ = −1.50 and −0.50) were significantly higher (*p* < 0.05) when compared separately to those displayed in other *CT*_*stop*_ conditions (except for *CT*_*stop*_ = 0.45). Concerning the high group, the crossing frequency observed for the lowest *CT*_*stop*_ value (*CT*_*stop*_ = 0.50) was significantly higher than in all conditions for which *CT*_*stop*_ ≥ 1.50 (*p* < 0.05, except for *CT*_*stop*_ = 2.00). No significant main effect of *CT*_*stop*_ was found for the inf group [*F*_(5, 45)_ = 0.30, *p* > 0.05]. Finally, no significant *CT*_*cross*_ × *CT*_*stop*_ interaction was found [*F*_(10, 90)_ = 0.55; *F*_(10, 90)_ = 1.42; *F*_(10, 90)_ = 1.75, *p* > 0.05 for the low, high and inf groups, respectively].

In sum, drivers attempted to cross the intersection more often, not only as *CT*_*cross*_ increased (for all three groups), but also as *CT*_*stop*_ decreased (for the low and high groups). As predicted, only the crossing affordance seemed to influence the inf group (where braking capabilities were infinite). Concerning the other groups, the increase in crossing frequency was higher in the low group (where the *CT*_*stop*_ was low) than the high group (where the *CT*_*stop*_ was higher). It seems that the frequency of crossing maneuvers results from a choice between crossing and stopping affordances (characterized by *CT*_*cross*_ and *CT*_*stop*_, respectively) for the low and high groups.

### Decision frequency: model

Based on our statistical results and mathematical constraints, we designed an *a posteriori* model able to quantify this choice between crossing and stopping affordances using the average inter-individual crossing frequency for the three groups. In the model, crossing frequency was only influenced by main effects of *CT*_*cross*_ and *CT*_*stop*_. Indeed, in the low and high groups crossing frequency increased with *CT*_*cross*_ and decreased with *CT*_*stop*_ with no significant *CT*_*cross*_ × *CT*_*stop*_ interaction. Consequently, for the low and high groups, the crossing frequency was modeled as a sum of two functions: an increasing *CT*_*cross*_ function, denoted *f*, and a decreasing *CT*_*stop*_ function, denoted *g*. The behavior of the inf group, which was assumed to be immune from choice between *CT*_*cross*_ and *CT*_*stop*_ affordances due to its infinite D_max_, was modeled differently. The ANOVA analysis of this group's behavior revealed a main positive effect of *CT*_*cross*_ but no main effect of *CT*_*stop*_. We thus modeled the crossing frequency of the inf group with a single increasing *CT*_*cross*_ function, denoted *f*. We used the same function *f* for the three groups as the group × *CT*_*cross*_ interaction was found to be non-significant.

The best mathematical formula found to describe *f* and *g* were sigmoid-type functions. Such functions were widely used to model verbal (Oudejans et al., 1996; Fajen and Matthis, 2011) and motor responses (Ishak et al., 2008) to affordances (see Franchak and Adolph, 2014 for a review about the use of probabilistic functions to model affordances; and Gescheider, 1997 for classical literature from which this habit was borrowed). A visual inspection of Figure 4 suggested that the negative effect of *CT*_*stop*_ on crossing frequency was exponential, with a plateau in crossing frequency for the lowest and highest *CT*_*stop*_ values. The change in crossing frequency as a function of *CT*_*stop*_ is given by the following formula: $g\left(CT_{stop}\right)=\frac{1}{1+e^{-b\cdot CT_{stop}}}$, where *b* ≤ 0 is a coefficient modulating the slope of the crossing frequency (i.e., the lower *b* the lower the slope of the sigmoid). We used a similar sigmoid formula to model the positive effect of *CT*_*cross*_ on crossing frequency: $f\left(CT_{cross}\right)=\frac{1}{1+e^{-a\cdot CT_{cross}}}$ where *a* ≥ 0 is the coefficient that modulates the slope of the crossing frequency. It should be noted that other mathematical functions would have been equally good candidates to fit this three point pattern. Moreover, in order to model the crossing frequency in the theoretical 0–100% range, another mathematical constraint was added. We modulated the action of *f* and *g* functions such that their sum never exceeded 100 using *w* and 1 − *w* weightings, with *w* ranging from 0 to 1. The choice between *CT*_*cross*_ and *CT*_*stop*_ on predicted crossing frequency was finally modeled as follows:

(1) $${\tilde{F}}_{cross}=100\times \left[\frac{w}{1+e^{-a\cdot CT_{cross}}}+\frac{1-w}{1+e^{-b\cdot CT_{stop}}}\right]$$

We used the Gauss–Newton algorithm to estimate the set of coefficients *a*, *b*, and *w* that minimize the sum of squares of residuals between the data and our model. With an identical set of coefficients for each group (*a* = 1.67, *b* = −1.69, and *w* = 0.57), the model offers a coefficient of determination equal to 0.84 and thus accurately reproduces our observations (Figure 4). The quality of this adjustment to our data can be further established by noting that this *R*^2^ is close to the maximal theoretical *R*^2^ equal to 0.92. This theoretical *R*^2^ can be computed using, as a model for crossing frequency, a sum of a polynomial of degree 17 in *CT*_*stop*_ (6 *CT*_*stop*_ modalities × 3 groups – 1, corresponding to the degrees of freedom related to *CT*_*stop*_) and a polynomial of degree 2 in *CT*_*cross*_ (3 *CT*_*cross*_ modalities −1, corresponding to the degrees of freedom related to *CT*_*cross*_). We considered the maximum theoretical value to be 0.92 as we did not observe a significant *CT*_*cross*_ × *CT*_*stop*_ interaction, theoretically explaining 8% of variance. Moreover, the low standard deviations of the coefficients (std = 0.56, 0.50, and 0.03 for *a*, *b*, and *w* coefficients, respectively) demonstrated their stability and confirmed that the model provided relevant predictions. A Student's *t*-test based on a *t*-distribution with *n* − *k* = 51 degrees of freedom (*n* and *k* refer to the number of points and the number of coefficients, respectively) tested the null hypothesis that each coefficient is equal to zero and demonstrated their significance.

First, the $\frac{1-w}{1+e^{-b\cdot CT_{stop}}}$ term accounts for the observed exponential increase in crossing frequency with *CT*_*stop*_, especially for the low group exposed to *CT*_*stop*_ values ranging from 1.75 to −1.50 s. The predicted crossing frequency exponentially increases as *CT*_*stop*_ values decrease until a maximum crossing frequency is reached when *CT*_*stop*_ values drop below −1.50 s. Second, this term is close to zero when *CT*_*stop*_ exceeds 2.00 s and no longer influences the crossing frequency. Therefore, in accordance with the statistical analysis, the crossing frequency model (Equation 1) can be reduced to a simpler formalization for the inf group exposed to *CT*_*stop*_ times higher than 2.50 s: ${\tilde{F}}_{cross}=100\times \left[\frac{w}{1+e^{-a\cdot CT_{cross}}}\right]$. Third, the $\frac{w}{1+e^{-a\cdot CT_{cross}}}$ term accounts for a similar increase in the modeled crossing frequency with *CT*_*cross*_ among the three groups. This is explained by their identical exposure to the three *CT*_*cross*_ times (0.00, 1.50, and 2.50 s) for a given *CT*_*stop*_ value.

In conclusion, the proposed model accurately reproduces our observations and predicts choice between *CT*_*cross*_ and *CT*_*stop*_ affordances for the average inter-individual crossing frequency. The model not only showed that manipulations of *CT*_*cross*_ induced changes in crossing frequency but also that manipulation of *CT*_*stop*_ modulated variation in crossing frequency. Finally, the model is able to account for the crossing frequency for all three groups with different action capabilities but identical coefficients. This supports the formalization of *CT*_*cross*_ and *CT*_*stop*_ as affordances, which are extrinsic properties of the intersection-crossing situations scaled to the driver's maximum acceleration and deceleration capabilities, respectively.

## Discussion

### Role of crossing and stopping possibilities on driver's decision frequency

Do driving maneuvers at the approach to an intersection emerge from choice between driving possibilities? Theoretically, drivers assess the viability of crossing the intersection based on traffic constraints and the acceleration of their car. However, at the same time, they assess the viability of performing a safe stop before the intersection based on their braking capability. Inspired by the original concepts of FST and MSZ introduced by Gibson and Crooks (1938), we formalized the viability of crossing as the critical time at which safe crossing is no longer possible (*CT*_*cross*_) and the viability of stopping as the critical time at which it is no longer possible to stop safely before the intersection (*CT*_*stop*_). In other words, *CT*_*cross*_ is the critical time at which the minimum satisfying acceleration for safe crossing (MSA) exceeds the driver's maximum acceleration (A_max_). In the same way, *CT*_*stop*_ is the critical time at which the minimum satisfying deceleration for safe crossing (MSD) exceeds the driver's maximum braking capabilities (D_max_). We tested the hypothesis that the decision taken by the driver is the result of choice between these crossing and stopping possibilities. We thus investigated the respective contribution of *CT*_*cross*_ and *CT*_*stop*_ to the decision taken by a driver when approaching an intersection in a driving simulator.

Three groups of participants drove cars with identical acceleration capabilities but different braking capabilities and were asked to try to cross an intersection before an oncoming car traveling orthogonally blocked the route. Alternatively, they could decide to stop before the intersection to let the oncoming car cross. Finally, as a last resort, they could decide to exit on the roadside. Since acceleration capability (A_max_) was constant for the three groups, between-group intersection-crossing situations offered identical crossing possibilities (*CT*_*cross*_). However, between-group differences in braking capabilities (D_max_) created different stopping possibilities (*CT*_*stop*_). We hypothesized that frequency of the decision to cross would increase as the viability of stopping decreased, although the viability of crossing was constant for all groups.

We consistently observed that crossing frequency increased as the critical time for crossing (*CT*_*cross*_) increased, irrespective of the group and the between-trial manipulation of *CT*_*stop*_. This result suggests that drivers perceive *CT*_*cross*_ as a variable that specifies the viability of crossing and underlines the driver's sensitivity to affordances that rely on maximum acceleration capabilities (Fajen and Matthis, 2011). Moreover, we found between- and within-group differences which showed that crossing frequency increased as *CT*_*stop*_ decreased. Although previous studies have shown that drivers are sensitive to affordances based on maximum deceleration capabilities, such as in a braking task (Fajen, 2005b,c), it is surprising to see the influence of braking capabilities on crossing decisions in our results. Finally, we found that the frequency of exiting increased when the combination of *CT*_*cross*_ and *CT*_*stop*_ ruled out crossing and stopping possibilities. Overall, these observations confirm that drivers take both the viability of crossing and stopping into account at the approach to an intersection.

### Potential perceptual processes underlying the driver's assessment of crossing possibilities

The influence of the car's braking capabilities on crossing frequency is the core result of this experiment. It raises questions about the mechanisms that combine crossing and stopping possibilities in making the final decision. What mechanism, underlying choice between the possibilities for action, could explain the influence of the driver's stopping possibilities on crossing frequency? In theory, all groups of drivers have an identical perception of their crossing possibilities, but our results show that in practice their stopping possibilities play a role in this assessment. Drivers appear to decide to cross an intersection not only because crossing is a very viable possibility, but also because stopping is not. This result is supported by our model, which suggests that drivers simultaneously assess crossing and stopping possibilities and weigh each of them. Moreover, our model is compatible with results from traditional affordance paradigms, as it explains behavior when drivers are exposed to a unique affordance (the inf group in our experiment). The following paragraphs evaluate candidate mechanisms which may explain these results.

We first ruled out a mechanism that consisted of the manipulation of the experimental instructions, i.e., drivers first assessed their crossing possibilities and then their stopping possibilities. This hypothesis would have led to similar between-group crossing frequencies for intersection-crossing situations where the safe crossing possibilities were identical. Moreover, stopping and exiting frequencies of all groups would have varied depending on stopping possibilities in situations with identical crossing possibilities. Although the observed stopping and exiting frequencies are compatible with this hypothesis, the different between-group crossing frequencies invalidate it.

It could be argued that the combined effect of crossing and stopping possibilities on crossing frequency is due to the fact that drivers do not perceive their crossing possibilities in the same way. In this case, a single affordance (perhaps including crossing and stopping possibilities) would sum up the decision-making process. Our implementations of critical times at which it is no longer safe to cross or stop in reference to the driver-intersection system are well-suited for the investigation of this kind of hypothesis. Indeed, considering that decision-making is based on critical times for both safe crossing and stopping possibilities is compatible with the idea that crossing possibilities are perceived in reference with the stopping possibilities. For instance, if *CT*_*cross*_ = 1.5 s and *CT*_*stop*_ = 3 s, these values will specify that safe stopping is afforded 1.5 s longer than safe crossing. Nevertheless, our statistical analysis, supported by a model that explains behavior given an identical set of *CT*_*cross*_ and *CT*_*stop*_ coefficients, suggests that these variables are separate affordances, among which drivers choose. In other words, drivers would identically perceive their possibilities to cross the intersection but would act in a different way depending on their stopping possibilities.

Alternatively, the final decision to select a given driving maneuver could be understood using a behavioral dynamics framework (Warren, 1998, 2006). In this framework, the final decision is consistent with a dynamic system. The possibility that offers the best chance of success among other available possibilities is the attractor for the final selection of a maneuver. On the other hand, the possibility that offers the least chance of success acts as a repeller in the final decision. This framework has been shown to account for route selection when steering, and avoiding obstacles in a complex environment (Fajen and Warren, 2003). Therefore, in our study, competing driving possibilities may act as the angular acceleration of the goal and the obstacle in the Fajen and Warren's steering task study and motivate the final decision as a function of their respective attractive or repulsive power. Further work needs to be carried out to confirm this hypothesis.

## Conclusion

Following Gibson and Crooks' footsteps, this experiment has shown that drivers not only take into account the crossing possibilities but also the stopping possibilities when performing cross or not decision at the approach of an intersection. Our results, supported by a behavioral model of decision making, provide an empirical support for the hypothesis of choice between affordances. Further research is needed not only to demonstrate the relevance of such a framework in the regulation of driving maneuvers (Fajen, 2005b), but also to evidence the optical correlates of *CT*_*cross*_ and *CT*_*stop*_.

## Conflict of interest statement

The authors declare that the research was conducted in the absence of any commercial or financial relationships that could be construed as a potential conflict of interest.
