# Supplementary material for: Drivers' decision-making when attempting to cross an intersection results from choice between affordances
Source: Front Hum Neurosci. 2015 Jan 9;8:1026. doi: 10.3389/fnhum.2014.01026 (PMC4288237; doi:10.3389/fnhum.2014.01026)
Supplement: Supplementary file 1 [file DataSheet1.DOCX]

***Supplementary Material***

**Drivers’ decision-making when attempting to cross an intersection results from choice between affordances**

Geoffrey MARTI^1^, Antoine H.P. MORICE^1*^ and Gilles MONTAGNE^1^

*Correspondence should be addressed to:

Antoine MORICE

Aix-Marseille Université, Faculté des Sciences du SportUMR 7287 “Institut des Sciences du Mouvement Etienne-Jules MAREY”
163 Avenue de Luminy
13288 Marseille cedex 09, France
Phone +33 491172273
Fax +33 491172252
(e-mail: antoine.morice@univ-amu.fr)

**Appendix**

**Reference frame**

The origin of the reference frame used to measure the position of the cars is located at the center of the two-lane road in abscissa and at the initial vertical position of the participant in ordinate (see Figure 2). The x-axis is positively oriented to the right and the y-axis is positively oriented upwards. The coordinates of the driver and the oncoming cars refer to the center of the vehicles.

**Initial kinematics of the oncoming car**

At t = 0, the x-axis coordinate of the oncoming car $x_{o}\left(0\right)$ corresponds to the distance to the center of the two-lane road. The y-axis coordinate of the oncoming car $y_{o}\left(0\right)$ refers to the distance between the participant’s car and the center of the perpendicular single lane road forming the intersection (see the initial distances of the participant’s car in Table 1). The oncoming car moves along the x-axis at a constant velocity $v_{o}\left(0\right)$ towards the intersection and stops when reaching the x-axis position of the participant’s car $x_{s}\left(0\right)$ in order to block the road. The variations of the x-axis coordinates of the oncoming car over time can thus be expressed as follows:

$x_{o}\left(t\right)=\left\{\begin{matrix} x_{o}\left(0\right)+v_{o}\left(0\right)\cdot t & if x_{o}\left(t\right)\leq x_{s}\left(0\right) \\ x_{s}\left(0\right) & otherwise \end{matrix}\right.$ (A1)

**Initial kinematics of the participant’s car**

At t = 0, the x-axis coordinate of the participant’s car $x_{s}\left(0\right)$ is equal to 1.75 meters (i.e., the position of the center of the right lane) and its y-axis coordinate $y_{s}\left(0\right)$ is equal to zero. The participant’s car initially moves along the y-axis at constant velocity $v_{s}\left(0\right)$. Before the participant’s manipulation of the pedals or the steering wheel, the variations of the y-axis coordinates of the participant’s car can thus be written as follows:

$y_{s}\left(t\right)=y_{s}\left(0\right)+v_{s}\left(0\right)\cdot t$ (A2)

**Equation of the CT_cross_**

CT_cross_ is defined as the last time at which the participant can initiate a maximum acceleration A_max_ in order to safely cross the intersection before the oncoming car. If a participant initiates a crossing maneuver with A_max_ at CT_cross_, his/her acceleration will be maintained constant until the end of the trial. The evolution of the driver’s velocity $v_{s}\left(t\right)$ can thus be written as follows:

$v_{s}\left(t\right)=v_{s}\left(0\right)+A_{\max}\cdot\left(t-CT_{cross}\right)$ $(t\geq CT_{cross})$ (A3)
The changes over time of the y-axis coordinates of the participant’s car $y_{s}\left(t\right)$ can be computed using the integral of the function $v_{s}$ from CT_cross_ to any t greater or equal to CT_cross_:

$y_{s}\left(t\right)-y_{s}\left(CT_{cross}\right)=\left(t-CT_{cross}\right)\cdot v_{s}\left(0\right)+\frac{\left(t-CT_{cross}\right)^{2}}{2}\cdot A_{\max}$ $(t\geq CT_{cross})$ (A4)
Substituting the y-axis coordinate $y_{s}\left(CT_{cross}\right)$ of the participant’s car at CT_cross_ with the initial kinematic of the participant’s car given in equation (A2) leads to:

$y_{s}\left(t\right)=y_{s}\left(0\right)+v_{s}\left(0\right)\cdot t+\frac{\left(t-CT_{cross}\right)^{2}}{2}\cdot A_{\max}$ $(t\geq CT_{cross})$ (A5)
At this stage, computing CT_cross_ requires to determine the time at which the oncoming car starts to endanger the safe crossing of the participant’s car. This occurs when the x-axis coordinate of the oncoming car’s front bumper is superior to the x-axis position of the left side of the participant’s car. This time is called TTC_o_ (Time to Contact of the oncoming car referred to the intersection). Since the oncoming car velocity is kept constant, TTC_o_ is defined as the ratio between the remaining x-axis distance before the oncoming car threatens safe crossing and the velocity of the oncoming car:

(A6)
where l_o_ (set to 4.41 meters) and w_s_ (set to 2.13 meters) are the length of the oncoming car and the width of the participant’s car, respectively. The definition of CT_cross_ implies that the y-axis position of the rear bumper of the participant’s car must coincide with the y-axis position of the left side of the oncoming car when t = TTC_o_, implying that the participant crossed the intersection at the latest possible moment. This leads to the following equation:

$y_{s}\left(TTC_{o}\right)=y_{o}\left(0\right)+\frac{l_{s}}{2}+\frac{w_{o}}{2}$ (A7)
where l_s_ (set to 4.41 meters) and w_o_ (set to 2.13 meters) are the length of the participant’s car and the width of the oncoming car, respectively. Using the equations (A5) and (A7), CT_cross_ satisfies the following equation in X:

$y_{s}\left(0\right)+TTC_{o}\cdot v_{s}\left(0\right)+\frac{\left(TTC_{o}-X\right)^{2}}{2}\cdot A_{\max}=y_{o}\left(0\right)+\frac{l_{s}}{2}+\frac{w_{o}}{2}$ (A8)
Finally, rearranging the expression (A8) gives that CT_cross_ is a root of the following second degree polynomial in X:

$\frac{A_{\max}}{2}\cdot X^{2}-\left(TTC_{o}\cdot A{}_{\max}\right)\cdot X+\frac{TTC_{o}^{2}\cdot A_{\max}}{2}+TTC_{o}\cdot v_{s}\left(0\right)-\left(y_{o}\left(0\right)+\frac{l_{s}}{2}+\frac{w_{o}}{2}-y_{s}\left(0\right)\right)$ (A9)

**Equation of the CT_stop_**

CT_stop_ is defined as the last time at which participant can initiate a maximum deceleration D_max_ in order to safely stop before the oncoming car. In the same vein of the CT_cross_ computation, the y-axis position and velocity of the participant’s car can be determined by considering an initiation of a stopping maneuver with a constant D_max_ at t = CT_stop_:

$v_{s}\left(t\right)=v_{s}\left(0\right)-D_{\max}\cdot\left(t-CT_{stop}\right)$ $(t\geq CT_{stop})$ (A10)
 $y_{s}\left(t\right)=y_{s}\left(0\right)+v_{s}\left(0\right)\cdot t-\frac{\left(t-CT_{stop}\right)^{2}}{2}\cdot D_{\max}$ $(t\geq CT_{stop})$ (A11)
According to the equation (A10), the time at which the participant’s car velocity becomes null $t_{v_{s}=0}$ is equal to:

$t_{v_{s}=0}=CT_{stop}+\frac{v_{s}\left(0\right)}{D_{\max}}$ (A12)
Based on the equation (A11), the y-axis position of the participant’s car can be written as follows:

$y_{s}\left(t_{v_{s}=0}\right)=y_{s}\left(0\right)+v_{s}\left(0\right)\cdot CT_{stop}+\frac{\left(v_{s}\left(0\right)\right)^{2}}{2\cdot D_{\max}}$ (A13)
The definition of CT_stop_ implies that the y-axis position of the front bumper of the participant’s car must coincide with the y-axis position of the right side of the oncoming car at t = $t_{v_{s}=0}$. This leads to:

$y_{s}\left(t_{v_{s}=0}\right)=y_{o}\left(0\right)-\frac{l_{s}}{2}-\frac{w_{o}}{2}$ (A14)
Using both equations (A13) and (A14) gives the following equation of CT_stop_:

$CT_{stop}=\frac{y_{o}\left(0\right)-\frac{l_{s}}{2}-\frac{w_{o}}{2}-y_{s}\left(0\right)}{v_{s}\left(0\right)}-\frac{v_{s}\left(0\right)}{2\cdot D_{\max}}$ (A15)
